# Can high-fidelity 3D models be a good alternative for cadaveric materials in skill assessment for endoscopic sinus surgery? A comparison study in assessment for surgical performance in 3D models and cadavers

**DOI:** 10.3389/fmed.2024.1301511

**Published:** 2024-10-17

**Authors:** Masanobu Suzuki, Ryosuke Watanabe, Akira Nakazono, Yuji Nakamaru, Takayoshi Suzuki, Shogo Kimura, Kotaro Matoba, Manabu Murakami, Dominik Hinder, A. J. Psaltis, Akihiro Homma, P. J. Wormald

**Affiliations:** ^1^Department of Otolaryngology-Head and Neck Surgery, Faculty of Medicine and Graduate School of Medicine, Hokkaido University, Sapporo, Japan; ^2^Department of Forensic Medicine, Faculty of Medicine and Graduate School of Medicine Hokkaido University, Sapporo, Japan; ^3^Center for Medical Education and International Relations, Hokkaido University, Sapporo, Japan; ^4^Department of Surgery–Otorhinolaryngology Head and Neck Surgery, Central Adelaide Local Health Network and the University of Adelaide, Adelaide, SA, Australia

**Keywords:** surgical education, 3D printer, cadaver surgery, surgical training, endoscopic surgery

## Abstract

**Introduction:**

Traditionally formal assessment of surgical skills has not been part of a surgeon’s accreditation process. The widely adopted apprentice model of “on-the-job training” does create additional risk for the patients. In the past surgical training has used cadavers, but these are expensive, require dedicated wet-lab facilities and are in increasingly short supply. In many countries religious and cultural practices also preclude cadaveric use. Recent 3D-printed technology allows mass reproduction of high-fidelity 3D models. In this study, we examined the utility of 3D sinus models compared to cadaver dissection for surgical skill assessment for endoscopic sinus surgery (ESS).

**Materials and methods:**

A total of 17 otolaryngologists performed Endoscopic Sinus Surgery (ESS) on 3D printed sinus models and then repeated these procedures on cadavers. Their surgical performance was assessed with the Objective Structured Assessment of Technical Skills (OSATS) score for ESS and time was taken to complete an ESS procedure. Their performance on the 3D models and cadavers was compared.

**Results:**

There were no significant differences in the OSATS score between 3D models and cadavers (50.41 ± 13.31 vs. 48.29 ± 16.01, *p* = 0.36). There was a strong positive correlation between the score in 3D models and those in cadavers (*r* = 0.84, *p* < 0.001). No significant differences were found in time for a mini-ESS (21:29 ± 0:10 vs. 20:33 ± 0:07, *p* = 0.53). There were positive correlations between 3D models and cadavers in time taken for a mini-ESS (*r* = 0.55, *p* = 0.04).

**Conclusion:**

The surgical performance on the 3D models was comparable to that on cadavers. This supports the utility of the 3D models as an inexhaustible alternative for cadavers in ESS surgical skill assessment.

## 1 Introduction

Surgery is a relatively high-risk activity and the training for surgery is often compared to that needed to fly commercial aircraft or manage a nuclear power station (1, 2). Most sinus surgery is performed for inflammatory or benign pathology to improve quality of life in individuals suffering from chronic rhinosinusitis (CRS). Endoscopic sinus surgery, a standard surgical treatment for medically resistant CRS (3), involves opening ostia and functional drainage units in the sinuses by resecting bony septations within the paranasal sinuses. Incomplete resection increases the risk of disease recurrence, while surgical error can result in severe complications given the close proximity of the sinuses to the orbit and the skull base. The efficacy and safety of ESS largely depends on surgeons’ surgical skills emphasizing the importance of the training of surgeons. Traditionally, such skills have been acquired by trainees through “on-the-job training.” Such a training model inevitably exposes patients to poorer surgical outcomes and a higher risk of complications, particularly during the steep learning curve of ESS surgery (4).

In other high-risk industries, such as aviation and nuclear power, simulation is used for training and also assessment of skills, with a certain level of demonstrated skills required before pilots or scientists are allowed to practice in the “real world” (4, 5). In the airline industry, a certain number of hours on the simulator are required not only during the training process but also as a part of routine certification and recertification throughout a pilot’s career (6, 7).

Similarly, in surgery, having such a credentialing process to assess all surgical trainees’ skills in simulation surgeries before initiating actual surgeries would be beneficial and potentially reduce the risks to the patients. To date, if surgeons wish to practice new techniques or learn/refine their skills, they can attend cadaveric courses. However, surgery on cadavers is not routinely as part of the surgical accreditation and licensing (8). This is in part due to limited availability and access to cadavers and wet lab facilities, the significant expense associated with their use and the fact that cadaveric anatomy is highly variable making standardized training/assessment impossible.

The advent of 3D technologies has been contributing to our daily life, also to many surgical specialties (9). Recent advances in 3D printing technology and polymer materials has paved the way for high fidelity 3D printed models with variations on complex anatomy produced from actual patient CT scans which create a set of challenging surgical situations through which surgical trainees can gain skills but which also allow an opportunity for standardized assessment of surgical skills (10). These models have very similar tactile feedback during surgery and have the elasticity of real mucosa and cartilage. Several studies have demonstrated that 3D anatomical models contribute to medical education for undergraduate students (11), optimization of surgical planning (12) and preoperative device selection (13) and of postoperative treatment (14). We recently reported on the usefulness of newly designed 3D sinus models in ESS skill acquisition and to estimate mental workload during surgeries (15, 16).

In this study, we examined the utility of the 3D sinus models as a medium in ESS skill assessment, by comparing ESS surgical performance for the 3D models and cadavers by surgical trainees.

## 2 Materials and methods

### 2.1 Participants

Data for this study was collected concurrently with the previously published studies regarding the validation of 3D-printed sinus models for ESS training (15). The institutional review board approved the present study (no. 018-043). A total of seventeen otolaryngologists in Hokkaido University Hospital voluntarily participated in the present study. The written informed consent was obtained from all the participants.

### 2.2 ESS for 3D models and for cadavers

Standard ESS instruments (Storz, Tuttlingen, Germany), with a 4-mm rigid nasal endoscope and a monitor (Telepac, Storz, Tuttlingen, Germany), and a powered microdebrider (Medtronic, Jacksonville, FL) were used.

**Figure 1:**
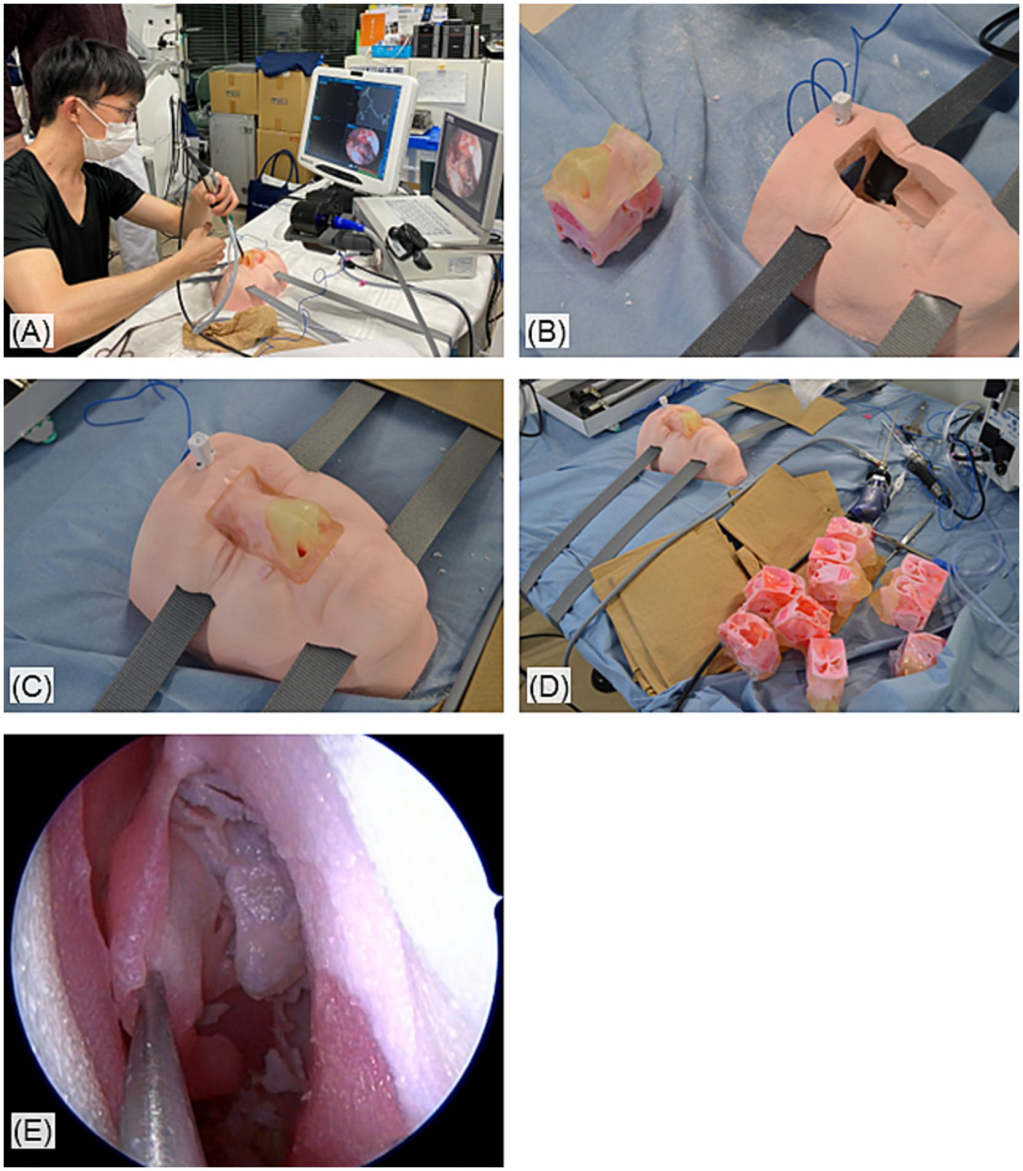
Overview of the simulation surgeries with the 3D-printed sinus models. **(A)** The setup of the simulation surgeries. **(B)** The 3D sinus models and its basement. **(C)** The models were inserted into and hold in the basement which mimics a human face. **(D)** The 3D models can be mass produced. **(E)** Endoscopic view of 3D-printed sinuses during the simulation surgeries.

Participants were allocated 45 min to perform full-house ESS (middle meatus antrostomy; MMA, anterior-and posterior-ethmoidectomy, sphenoidotomy, and frontal sinusotomy) on the left side of 3D-printed sinus models (PJW-N2-V2, Fusetec, Adelaide, South Australia), printed from the dicoms of CT scans of actual patients with chronic rhinosinusitis (Figure 1). The validation of the 3D models and the mock surgeries with them for ESS simulation is reported in detail in the previous paper (15). To evaluate the progress within the allocated time under the standardized condition, the order of the surgical steps to be performed, as well as the surgical instrument to be used in each step, was set and performed in the following order, uncinectomy, MMA, anterior-and posterior-ethmoidectomy, sphenoidotomy, and finally, frontal sinusotomy. Within 48 h after their 3D model surgeries, participants performed full-house ESS for cadavers with the same equipment and under the same conditions. All surgeries were video recorded (15).

### 2.3 Assessment of surgical performance

Participants’ surgical performance were assessed with the Objective Structured Assessment of Technical Skills (OSATS) score for ESS (17). Briefly, the scoring system was designed for evaluation of the performance during every step in ESS beginning with handling the endoscope and intranasal preparation going all the way to perform a frontal sinusotomy. The whole process in ESS is subdivided into 21 tasks, and each task is evaluated with a 5-point Likert rating scale with “unable to perform” for 1, “able to perform majority” for 3, and “performs easily with good flow” for 5, respectively (Table 1). In the present study, the scoring for three tasks in the intranasal preparation were excluded because vasoconstrictor and local anesthesia was not required for 3D models and cadavers; the remaining 18 tasks were evaluated with the perfect score of 90. The OSATS score was blindly assessed by other two rhinologists (MS and YN) based on the recorded video. High inter-rater reliability has been confirmed, and the detail is described in previous paper (15).

**Table 1:** The scoring system used in this study that was modified from the Objective Structured Assessment of Technical Skills (OSATS) score for ESS originally reported by Lin et al. (17).

Tasks	Unable to perform		Able to perform majority		Performs easily w/good flow
Sinus endoscopy					
Inferior pass	1	2	3	4	5
Intermediate pass	1	2	3	4	5
Superior pass	1	2	3	4	5
Uncinectomy					
Identification of uncinate and boundaries	1	2	3	4	5
Incision of uncinate with backbiter or sickle knife	1	2	3	4	5
Removal of uncinate with forceps or debrider	1	2	3	4	5
Maxillary antrostomy					
Identification of natural ostium of maxillary sinus	1	2	3	4	5
When indicated, enlargement of ostia by removal of posterior fontanelle	1	2	3	4	5
Anterior ethmoidectomy					
Identification of bulla	1	2	3	4	5
Removal of bulla with mucosal preservation with forceps or debrider	1	2	3	4	5
Removal of anterior cells with identification of boundaries (middle turbinate, basal lamella, lamina papyracea)	1	2	3	4	5
Posterior ethmoidectomy					
Low entrance into basal lamella with preservation of horizontal strut	1	2	3	4	5
Removal of posterior ethmoid cells with identification of skullbase, superior turbinate	1	2	3	4	5
Sphenoidostomy					
Entrance via posterior ethmoids at inferiomedial triangle or entrance thru natural ostium	1	2	3	4	5
Enlargement of sphenoid ostia	1	2	3	4	5
Demonstration of internal carotid, optic nerve location	1	2	3	4	5
Frontal sinusotomy					
Atraumatically removes bony partitions in the frontal recess	1	2	3	4	5
Defines the skull base and orbital wall	1	2	3	4	5

Progress status of surgeries was evaluated with the surgical procedures that the participants had completed by the end of the allocated time. For statistical analysis of procedures, the status was converted to the following number (1: none, 2: uncinectomy, 3: MMA, 4: anterior-ethmoidectomy, 5: posterior-ethmoidectomy, 6: sphenoidotomy, 7: frontal sinusotomy and completion of full-house ESS). For example, if a participant had finished posterior-ethmoidectomy and was performing a sphenoidotomy when time was up, the progress was expressed as “3: posterior-ethmoidectomy.” The time taken to complete a mini-ESS (MMA and anterior ethmoidectomy) was also recorded (15). The reason to select a mini-ESS is that the maxillary sinus and anterior ethmoid anatomy are consistent between cadavers, but frontal sinus anatomy is highly variable, which would make it very difficult to compare a frontal sinus dissection between the model and a cadaver.

### 2.4 Statistical analysis

Shapiro–Wilk tests were applied to evaluate if the data fitted a normal distribution curve. Parametric data and nonparametric data were expressed as mean (±SD) and median with the interquartile range, respectively. Parametric data were assessed with a paired, two-tailed t-test for comparison between surgical performances in 3D models and in cadavers. Similar, nonparametric data were assessed with Wilcoxon test. Pearson (for parametric data) or spearmen coefficients analysis (for nonparametric data) were used to assess correlation between them. *p* values of less than 0.05 were considered statistically significant. All the analyses were performed by using JMP 11 (SAS Institute Inc.).

## 3 Results

### 3.1 Characteristics of the participants and the assessors

The experienced years of the participants and the assessors as otolaryngologists were 2.7 ± 4.0 and 23.0 ± 4.0, respectively. The previous experienced ESS cases of the participants and the assessors were 41.2 ± 117.6 and 700.0 ± 424.3, respectively. Two out of the participants and both of the assessors were officially certified board members of the Japanese otolaryngology society. All the participants belonged or had belonged to the Department of Otolaryngology, Hokkaido University Hospital, where the assessors worked for. None of the participants hold a conflict of interest to participate in this study. Both assessors hold instructor licenses certified by the Japanese otolaryngology Society.

### 3.2 Comparison on the surgical quality assessment between 3D models and cadavers

**Figure 2:**
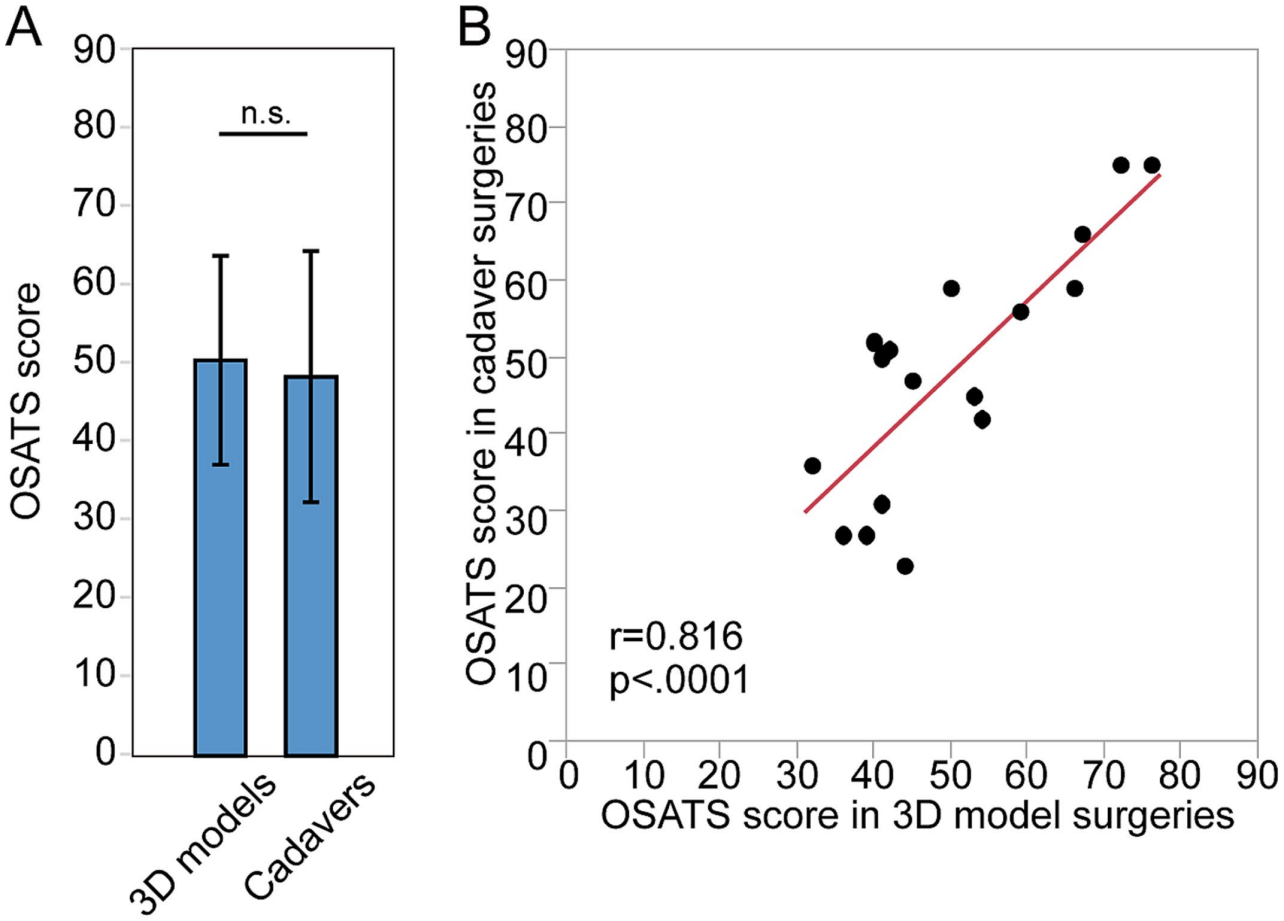
Quality assessment of surgeries done by the same individual surgeons for 3D models and for cadavers. **(A)** The OSATS score of surgeries done for 3D models and for cadaver. **(B)** Correlation between the OSATS score in 3D models and in cadaver.

A total of 17 surgeries for 3D models and their paired surgeries for cadavers were assessed (Table 2). The OSATS score in 3D models and cadaver was 50.41 ± 13.31 and 48.29 ± 16.01, respectively (Table 2; Figure 2A). There was no significant difference between them (*p* = 0.36). There was significant positive correlation between OSATS score in 3D models and cadaver (*r* = 0.82, *p* < 0.0001, Figure 2B).

**Table 2:** Characteristics of surgeries for 3D models and cadavers.

	Surgeries for 3D models (*n* = 17)	Surgeries for cadavers (*n* = 17)	*p* value
OSATS score	50.41 ± 13.31	48.29 ± 16.01	0.36
Progress status			
Progress of surgeries (median, IQR)	5 (1.5–4.5)	5 (4–6)	0.48
7: Frontal sinusotomy (*n*, %)	3 (17.65%)	2 (11.76%)	
6: Sphenoidotomy (*n*, %)	4 (23.53%)	4 (23.53%)	
5: Posterior ethmoidectomy (*n*, %)	6 (35.29%)	5 (29.41%)	
4: Anterior ethmoidectomy (*n*, %)	3 (17.65%)	4 (23.53%)	
3: MMA (*n*, %)	1 (5.88%)	1 (5.88%)	
2: Uncinectomy (*n*, %)	0 (0%)	1 (5.88%)	
1: None (*n*, %)	0 (0%)	0 (0%)	
Spending time			
Time taken for a mini-ESS (sec)	21:29 ± 0:10	20:33 ± 0:07	0.53

OSATS: Objective Structured Assessment of Technical Skills, IQR: interquartile range, MMA: Middle meatus antrostomy, Mini-ESS: middle meatal antrostomy and anterior ethmoidectomy.

### 3.3 Assessment on the efficiency of surgeries

**Figure 3:**
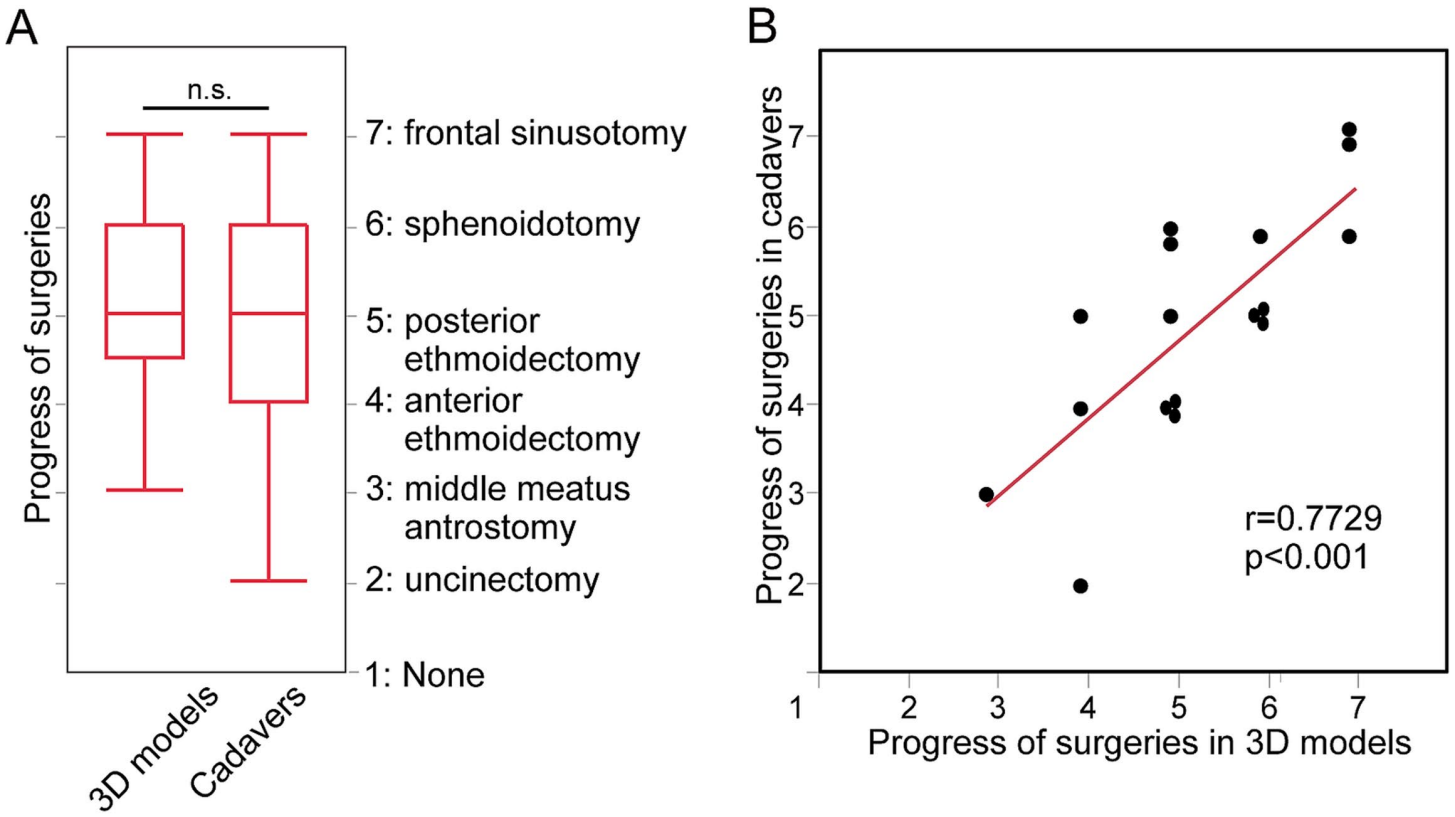
The progress status of surgeries done by the same individual surgeons for 3D models and for cadavers. **(A)** The progress status of surgeries done for 3D models and for cadavers. **(B)** Correlation between the status in 3D models and in cadavers.

As for the progress status of surgeries, there were no significant differences in 3D models and cadavers (*p* = 0.48, Figure 3A). Significant positive correlation in progress status were found between 3D models and cadavers (*r* = 0.77, *p* < 0.01, Figure 3B).

**Figure 4:**
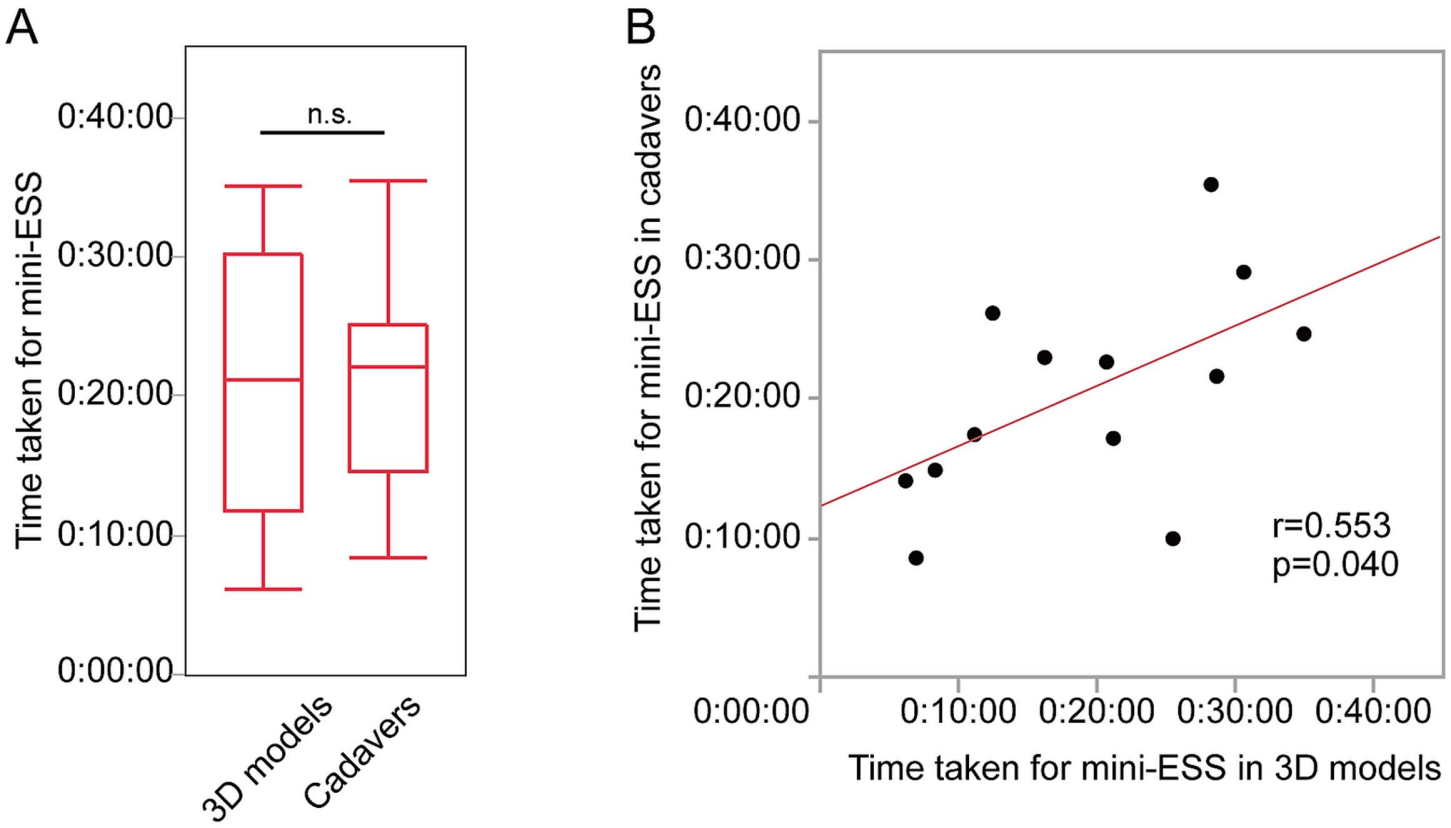
The time taken for mini-ESS by the same individual surgeons for 3D models and cadavers. **(A)** The time taken for mini-ESS done on 3D models and for cadavers. **(B)** Correlation between the time for a mini-ESS in 3D models and in cadavers.

There were no significant differences in spending time for a mini-ESS (3D models 21:29 ± 0:10 and cadavers 20:33 ± 0:07, *p* = 0.53, Figure 4A). The time taken to complete a mini-ESS in 3D models were significantly positively correlated to the time taken in cadavers (a mini-ESS *r* = 0.553, *p* = 0.0402, Figure 4B).

## 4 Discussion

This study examines the use of 3D models compared to cadavers as an assessment tool for surgeons performing ESS. Overall, we observed that the surgical performance of dissectors on 3D models was similar to that seen on cadavers. There was a high level of correlation between the 3D printed model and the cadaver for both quality (OSATS score) of the dissection as well as surgical progression through the dissection steps. The significant benefit of the 3D models is the high level of similarity in tactile feedback and similarity of the surgical experience when dissecting mucosa and bony septations. Unlike a cadaver, the 3D models allow for repetitive training on a model until the acquisition of a surgical skill has been attained and also facilitates for a graduated learning experience by allowing models of different degrees of anatomical complexity to be printed. 3D models are also able to be mass produced allowing for both surgical skill level development and standardized assessment of trainee’s surgical skill.

To date there have been various 3D-printed models developed in the field of otolaryngology (18–31) with most involving the temporal bone. To date there is only one study where 3D printed models have been compared to cadavers (32). In this study as a part of the validation of their 3D sinus models, they found a positive correlation in their ESS score between their 3D models and cadavers. This study focused on the validation of the model rather than on their utility as an assessment medium, and they did not investigate surgical efficiency either (32).

Obtaining a sufficient supply of cadavers has been problematic in many countries around the world (33, 34). If training on cadavers were introduced as a prerequisite to trainees performing surgery on patients, it is estimated that at least 100–150 cadavers per year would be necessary for assessment for ESS skills in Japan. This is an unrealistic number even though Japan is one of the most successful countries to establish highly successful body donation programs for surgical education (33). Furthermore donated cadavers are often unsuitable for ESS training due to prior surgery or disease presence (29.2% of the total cadavers assessed in this study were unsuitable). During this study some trainees, who were assigned cadavers found that the cadavers had already undergone surgery, had distorted anatomy/fractures due to the handling and storage process or were contaminated. These factors were perceived by surgeons to have negatively affected their surgical skill acquisition.

3D models have many advantages over cadavers (15, 35). These include a lack of regulatory and ethical issues with regards to their acquisition, use and disposal. Furthermore, they can be produced en mass with reliable, and predictable anatomy with variable levels of difficulty which make them ideal for the graduated training of surgeons of different levels. It should be noted that disadvantages do exist. These include tissue feel and handling and the lack of neighboring anatomy such as the neurovascular structures, the orbit, brain and infra-temporal fossa. With this said, as polymer technology continues to evolve and improve, many of these limitations are being addressed with the gap closing between cadavers and 3D models.

The utility of 3D models in surgical training and assessment as an alternative to cadavers, as demonstrated in this study, could be developed for other surgical disciplines. Several anatomical models have already been developed for the fields of orthopedic, neurology, and gynecology. Also, same methodologies could be applied to Endonasal Endoscopic Odontoidectomies for cranio-cervical junction pathologies (36), as the surgeries hold similarities with ESS.

There are several limitations to this study. Due to the rarity of cadavers, the number of analyzed subjects was limited. The numbers of surgical trainees undertaking this study was limited, complications occurring in the dissections were not recorded and it is unknown if the skill acquired directly translated to better surgical performance in patients. Ideally, a randomized controlled study will be necessary where surgical performance in actual surgery should be compared to those in 3D models. Despite these limitations, this pilot study illustrates the utility of 3D models as a promising alternative tool to both train surgeons and to assess skill acquisition for ESS accreditation.

## 5 Conclusion

The surgical performance of trainees in 3D sinus models was similar to that seen in cadavers, which supports the utility of the 3D models in ESS surgical skill training and assessment. With the lack of ethical concerns, the ability to conduct the assessment in any facility, predictability, reproducibility of anatomy for the simulation, and a lack of infectivity, 3D models can be an inexhaustible alternative for cadavers in ESS surgical skill assessment.

## Data Availability

The raw data supporting the conclusions of this article will be made available by the authors, without undue reservation.
